# The Association between Problematic Smartphone Use and Mental Health in Austrian Adolescents and Young Adults

**DOI:** 10.3390/healthcare12060600

**Published:** 2024-03-07

**Authors:** Doris Mayerhofer, Katja Haider, Manuela Amon, Afsaneh Gächter, Teresa O’Rourke, Rachel Dale, Elke Humer, Thomas Probst, Christoph Pieh

**Affiliations:** 1 Department of Psychosomatic Medicine and Psychotherapy, University for Continuing Education Krems, 3500 Krems an der Donau, Austria; 2 Faculty of Psychotherapy Science, Sigmund Freud University Vienna, 1020 Vienna, Austria; 3 Division of Psychotherapy, Department of Psychology, Paris Lodron University of Salzburg, 5020 Salzburg, Austria

**Keywords:** problematic smartphone use, loneliness, physical inactivity, mental health, adolescents, young adults

## Abstract

Although problematic smartphone use (PSU) is prevalent and associated with mental health and physical activity, there are no studies on its prevalence and associations in Austria. The aim of this study was to evaluate the prevalence of PSU and its associations with mental health in adolescents and young adults. A cross-sectional online survey was performed from 19 April to 27 July 2023, and the data of N = 913 respondents (14.1% male, 82.4% female, 3.5% diverse; median age: 17 [IQR: 15–18]; range: 14–20 years) were included in the analyses. Overall, 38.1% (females: 39.0%, males: 33.3%) of those surveyed were above the cut-off for PSU measured with the Smartphone Addiction Scale (SAS-SV). In addition to screen time, PSU is also associated with depressive symptoms (aOR = 1.46), anxiety symptoms (aOR = 1.86), disordered eating (aOR = 1.55), and alcohol abuse (aOR = 1.71), but not physical inactivity. On the other hand, physical inactivity was associated with depressive symptoms (aOR = 2.48), anxiety symptoms (aOR = 1.74), distress (aOR = 2.02), and low well-being (aOR = 3.25). A total of 37.7% respondents reported being strongly lonely, as measured with the De Jong Gierveld Loneliness Scale. The amount of screen time, but not PSU, was associated with loneliness. In sum, PSU affects more than one-third of adolescents and young adults in Austria and is associated with increased mental health symptoms. However, intensive screen time seems to be more strongly associated with increased mental health symptoms than PSU itself. The study confirms once again that smartphone use is associated with negative effects and that they should be used responsibly.

## 1. Introduction

Since anxiety and depression were among the top 10 Global Burdens of Diseases among adolescents and young adults aged 10 to 24 years in 2019 [1], the potential of health-related behaviors in terms of prevention and therapy needs to be emphasized. In this regard, the use of smartphones constitutes a health-related behavior that has a considerable role within the public health discourse. Besides the importance and usefulness of smartphones in everyday lives nowadays, the potential of disadvantageous usage patterns should not be neglected. Problematic smartphone use (PSU) seems to be rather prevalent worldwide. According to a meta-analysis, the estimated global prevalence of smartphone addiction was 26.99%, and for the included European region, the prevalence rate was 18.51% [2]. Concerning young people, a systematic literature review and meta-analysis reports that PSU is prevalent in approximately every fourth child or young person [3]. PSU and smartphone addiction are linked with the amount of daily smartphone usage [4,5,6].

There are manifold implications of smartphone usage as regards health-related behavior. For instance, dysfunctional smartphone use has repeatedly been associated with negative mental health outcomes. Since its association with depression, anxiety, sleep disturbances, and distress [3,6,7,8], the high prevalence rates of PSU should be a cause for concern. Moreover, studies have revealed associations of dysfunctional smartphone usage patterns with increased feelings of loneliness or social isolation [9,10]. Social isolation and loneliness are both associated with an increased risk of mental health problems in children, adolescents, and adults [11,12,13]. Furthermore, problematic aspects of excessive amounts of smartphone use are also related to sedentariness [14,15,16] and decreased physical activity and fitness [17,18,19].

In Austria, study reports indicate an increase in the amount of daily smartphone usage throughout the past few years [20,21]. Prior surveys including Austrian adolescents or a sample representative of the general population also revealed associations between health-related behavior like the daily amount of smartphone usage or physical activity and depression, distress, anxiety, and insomnia [20,22]. Regarding smartphone usage, a positive association with anxiety, distress, depression, and insomnia was found [20,21,22]. At a minimum of five hours of smartphone usage per day, the adjusted odds ratios (aORs) of reporting relevant mental health problems were significantly higher for all indicators in comparison to participants reporting a maximum of one hour per day [20]. Furthermore, the odds of reporting clinically relevant symptoms of depression were significantly lower in respondents who reported at least one physically active day throughout the last week prior to the survey compared to inactive respondents. Additionally, the accumulating number of physically active days per week was associated with a gradual decrease in the odds of being screened positive for depression [20]. These prior surveys have resulted in an association between the amount of smartphone usage or physical activity and mental health indicators. However, they used a single item to examine smartphone usage by asking about the daily amount of smartphone screen time (hours/day) and did not use a standardized instrument to assess PSU.

Our online survey was conducted between 19 April and 27 July 2023 and set out to assess the association between the daily amount of smartphone use, PSU, and physical inactivity and the mental health of Austrian adolescents and young adults aged 14 to 20 years. Our respondents were asked to answer mental health questionnaires in order to assess depressive symptoms, anxiety symptoms, insomnia symptoms, symptoms of alcohol abuse, and symptoms of disordered eating, as well as perceived distress, well-being, and loneliness. To explore health-related behaviors, our respondents also answered the short version of the Smartphone Addiction Scale (SAS-SV) [23] as a standardized measure of PSU, and we asked our respondents about their daily amount of smartphone usage and their physical activity.

Our first research question addressed the prevalence of PSU within the sample of Austrian adolescents and young adults. Secondly, we aimed to assess the association between demographic variables and the likelihood of being categorized as being smartphone addicted by the SAS-SV. The main objective of our current survey was to examine the association between health behaviors like the daily amount of smartphone usage, PSU, and physical inactivity and mental health outcomes. More specifically, we aimed to examine if the likelihood of reporting clinically relevant symptoms in any mental health questionnaire is associated, firstly, with the daily amount of smartphone use; secondly, with PSU; and thirdly, with physical inactivity. We tested the hypothesis that demographic variables (gender, age, education, migration background, the daily amount of smartphone usage, and physical inactivity) are associated with an increased likelihood of being categorized as smartphone addicted by the SAS-SV. We further hypothesized that the daily amount of smartphone use, PSU, and physical inactivity are associated with an increased likelihood of reporting mental health problems. The current report summarizes the results of our cross-sectional study and illustrates the association between these variables. Since our results suggest an association between the examined health behaviors and mental health indicators, the public health relevance of the amount of smartphone use, PSU, and physical inactivity are discussed.

## 2. Materials and Methods

### 2.1. Ethical Considerations

The current study was approved by the data protection officer and by the Ethics Committee of the University for Continuing Education Krems, Austria (protocol code: EK GZ 41/2018–2021). The procedure followed the Declaration of Helsinki. All respondents agreed to the data protection declaration and gave informed consent. Since the minimum legal age of consent is 14 years in Austria, our respondents also had to confirm a minimum age of 14 years before they started to work through the survey.

### 2.2. Study Design

The online survey was carried out as a cross-sectional study and aimed to examine the mental health status of Austrian pupils and students in the age range of 14 to 20 years and its association with physical inactivity and the daily amount of smartphone use. Data were collected between 19 April and 27 July 2023. The recruitment strategy involved online convenience sampling, with school representatives being contacted and asked to share an invitation containing the link to the online survey on various social media platforms for students. The online survey was conducted via Lime Survey (LimeSurvey GmbH, Hamburg, Germany).

### 2.3. Sample Characteristics

From an initial sample of N = 1961 eligible respondents, n = 784 did not answer the whole survey and therefore were excluded from the final analyses. From the remaining respondents (N = 1177), we further excluded individuals who reported to be older than 20 years of age (n = 236) or who reported that they did not attend any kind of education at the time of their participation (n = 28). The data of N = 913 respondents were included in the analysis. The final sample comprised 752 (82.4%) female, 129 (14.1%) male, and 32 (3.5%) diverse respondents (i.e., respondents who did not identify as a man or woman). The median (Med) age was 17, and the interquartile range (IQR) was 15 to 18. A first- or second-generation migration background was reported by 163 (17.9%) respondents. Table 1 further illustrates the characteristics of the study sample.

### 2.4. Instruments

#### 2.4.1. Depression

Depressive symptoms were assessed via the Patient Health Questionnaire 9 (PHQ-9). A cut-off score indicates clinically relevant suffering from depressive symptoms. Adults are screened positive for depression with a PHQ-9 sum score of 10 or more; in adolescents (<18), the recommended cut-off value is 11 [24,25]. For PHQ-9, Cronbach’s α was 0.867 in the current sample.

#### 2.4.2. Anxiety

The GAD-7 (General Anxiety Disorder 7) questionnaire was used to assess clinically relevant symptoms of anxiety. Adults are screened positive for a relevant level of anxiety with a sum score of 10 and above, and a cut-off score of 11 is applied for adolescents (<18) [26,27]. In the current sample, Cronbach’s α was 0.875 for the GAD-7.

#### 2.4.3. Insomnia

The Insomnia Severity Index (ISI) was used to examine problems related to the respondents’ sleep. The questionnaire comprises seven items. The evaluation gives a sum score ranging from 0 to 28. A cut-off value of 15 corresponds to moderate insomnia and screens positive for relevant sleep disturbances [28]. Cronbach’s α was 0.785 for the ISI.

#### 2.4.4. Alcohol Abuse

The CAGE (Cut Down, Annoyance, Guilt, Eye-Opener) questionnaire [29] is a frequently used and well-established screening tool to assess problematic alcohol use. Four items aim to detect alcohol abuse. The total score ranges from 0 to 4; a sum score of two and above indicates alcohol abuse [30]. Within the current sample, Cronbach’s α was 0.519.

#### 2.4.5. Eating Disorders

The SCOFF (Sick, Control, One Stone, Fat, Food) questionnaire is intended to serve as a quick screener of disordered eating [31,32]. It comprises five items, and the total score ranges from 0 to 5. A sum score of two and above serves as a cut-off value and indicates disordered eating. The current sample yielded a Cronbach’s α of 0.559.

#### 2.4.6. Distress

The Perceived Stress Scale 4 (PSS-4) was used to assess the perception of distress in the current sample [33,34]. It comprises four items and results in a sum score which ranges from 0 to 16. As we used binary logistic regression models to analyze our data, a median split was applied to dichotomize the PSS-4 outcomes in order to include them in the analyses. We obtained two categories, 0 = “lower levels of distress” and 1 = “higher levels of distress” (Med = 9 [IQR: 6; 12]). Cronbach’s α was 0.816.

#### 2.4.7. Well-Being

Aiming to assess the respondents’ well-being, the German version of the WHO-5 Index [35,36] was used. The evaluation of the questionnaire results in a sum score that ranges from 0 (indicating no well-being) to 25 (indicating high well-being). A multiplication by 4 translates the sum score into a percentage scale, ranging from 0 to 100. We applied a median split to the percent scores of WHO-5 to include the questionnaire in our binary logistic analysis (Med = 36 [IQR: 22; 52]), resulting in two distinct categories: 0 = “lower levels of well-being” and 1 = “higher levels of well-being”. For the WHO-5, Cronbach’s α was 0.815.

#### 2.4.8. Problematic Smartphone Use (PSU)

We used the short version of the Smartphone Addiction Scale (SAS-SV) [23] as a measure of PSU. The questionnaire comprises 10 items and was validated in German [4]. It results in a total score which ranges between 10 and 60. Cut-off scores have been established for males, who are considered smartphone addicted at a score of 31 and above, and females, who are considered smartphone addicted at a score of 33 and above [23]. Hence, analyses including the SAS-SV did not include the data of diverse respondents. Cronbach’s α for the SAS-SV was 0.833.

#### 2.4.9. Loneliness

To assess loneliness, we used the De Jong Gierveld Loneliness Scale [37]. The scale comprises 11 items: six items assess the dimension of emotional loneliness, and five items assess social loneliness. The evaluation results in a total score ranging from 0 to 11. Cut-off values can be used to differentiate between not (0–2), moderately (3–8), and strongly (9–11) lonely. We set the cut-off at 9 to differentiate between respondents who described themselves as not or as moderately lonely and those who described themselves as strongly lonely. Cronbach’s alpha was 0.884 for the items of the social loneliness subscale and 0.807 for the emotional loneliness subscale.

#### 2.4.10. Other Variables

The variable Physical Activity was assessed with a single question asking about the number of active days in the week before the survey. Like previous studies [38,39], we also defined physical activity as a minimum of 60 min/day on at least one day per week up to a maximum of seven days per week. Physical inactivity was defined as zero active days per week. Sixty minutes of physical activity per day were set as a reference due to international recommendations on physical activity in adolescents [40]. Smartphone use was also assessed with a single question asking about the daily amount of smartphone use. The respondents were provided with a scale to rate their daily smartphone usage: <1 h, 1–2 h, 3–4 h, 5–6 h, 7–8 h, and >8 h. Previous studies have also used these categories to operationalize smartphone use [38,39]. Since only 1.3% reported using their smartphone for less than one hour per day, we decided to combine the first two categories (0–2 h). All duration categories (3–4 h/day, 5–6 h/day, 7–8 h/day, and ≥8 h/day) were compared to the reference category of respondents who reported 0–2 h/day. The reference category is also referred to as “low-users”.

### 2.5. Statistical Analysis

Data analysis was carried out using the statistic software IBM SPSS Statistics Version 27 (IBM Corp., Armonk, NY, USA). The mental health outcomes were dichotomous (i.e., cut-off values: non-significant vs. clinically significant outcomes; median split: lower vs. higher levels of well-being or distress). Therefore, multivariable binomial logistic regression analyses were performed to examine the associations between the predictor and outcome variables. To examine the association of PSU with sociodemographic variables, a multivariable binary logistic regression was used. Odds ratios were adjusted for age, gender (female vs. male), migration background (yes vs. no), education, smartphone usage (hours/day), physically active days per week (active vs. inactive), and loneliness (not lonely, moderately lonely, or severely lonely). Another multivariable binomial logistic regression model was used to explore the association of smartphone use (five categories) and physical inactivity (two categories) with mental health outcomes. Regarding physical inactivity and smartphone use, odds ratios were adjusted for age, gender (female, male, or diverse), migration background (yes vs. no), education, smartphone usage (hours/day), and physical inactivity (active vs. inactive) (N = 913). A further binary logistic regression model which also included the SAS-SV outcomes as a measure of PSU (two categories) (N = 881) was used. Odds ratios were adjusted for age, gender (female or male), migration background (1st or 2nd generation; yes vs. no), education, smartphone usage (hours/day), physically active days per week (active vs. inactive), and PSU (below cut-off vs. cut-off and above). Binary logistic regression analysis resulted in adjusted odds ratios (aORs) and their 95% confidence intervals (±95%CIs). The ±95%CIs were estimated to display statistical uncertainty. The significance level was set at a *p*-value < 0.05 (2-sided tests).

## 3. Results

Table 1 illustrates the sample characteristics. The right-hand side of Table 1 summarizes the results of binary logistic regression analyses to explore associations of demographic variables, smartphone usage, physical inactivity, and loneliness with SAS-SV cut-off scores. The aORs are presented for age (aOR = 0.92; 95%CI: 0.83–1.03), gender (male compared to female) (aOR = 0.92; 95%CI: 0.60–1.41), migration background (first or second generation; yes compared to no) (aOR = 1.01; 95%CI: 0.69–1.49), education (academic secondary school [aOR = 1.09; 95%CI: 0.78–1.51]; university/university of applied sciences [aOR = 1.69; 95%CI: 0.92–3.10]; [pre-/intermediate] vocational education or apprenticeship [aOR = 0.55; 95%CI: 0.30–1.02]; or other [aOR = 1.30; 95%CI: 0.65–2.62] compared to higher vocational education), physical inactivity (compared to physical activity) (aOR = 1.04; 95%CI: 0.67–1.60), loneliness (moderately lonely [aOR = 1.13; 95%CI: 0.73–1.75] or strongly lonely [aOR = 1.26; 95%CI: 0.80–1.99] compared to not lonely) and the daily amount of smartphone use (3–4 h/day, 5–6 h/day, 7–8 h/day, or ≥8 h/day compared to 0–2 h/day). The daily amount of smartphone use is the only demographic variable which was associated with the likelihood of being categorized as smartphone addicted by the SAS-SV. AORs were 3.59 (95%CI: 1.96–6.60) for 3–4 h/day, 6.69 (95%CI: 3.76–12.89) for 5–6 h/day, 13.38 (95%CI: 6.55–27.36) for 7–8 h/day, and 9.85 (95%CI: 4.74–20.46) for more than 8 h/day compared to 0–2 h/day of smartphone usage.

Table 2 summarizes the results of binary logistic regression analyses. The aORs of reporting mental health problems, higher levels of distress, low well-being, and loneliness across the mental health indicators are presented for physical inactivity (compared to physical activity), PSU (below cut-off vs. cut-off and above), as well as smartphone use (3–4 h/day, 5–6 h/day, 7–8 h/day, or ≥8 h/day compared to 0–2 h/day).

Within the group of respondents who reported being physically inactive, the aORs of clinically relevant symptoms of depression (aOR = 2.48 [95%CI: 1.48–4.16]), anxiety (aOR = 1.74 [95%CI: 1.13–2.68]), high levels of distress (aOR = 2.02 [95%CI: 1.32–3.10]), and low well-being (aOR = 3.25 [95%CI: 2.03–5.21] were significantly higher than in respondents who reported being physically active (1–7 days/week, a minimum 60 min).

Regarding PSU (n = 881), 336 (38.1%) respondents were identified as being smartphone addicted by the SAS-SV (female: n = 293 [39.0%]; male: n = 43 [33.3%]). The odds of being screened positive for depression (aOR = 1.46 [95%CI: 1.06–2.01]), anxiety (aOR = 1.86 [95%CI = 1.37–2.51], disordered eating (aOR = 1.55 [95%CI: 1.13–2.11]), and alcohol abuse (aOR = 1.71 [95%CI = 1.19–2.45]) were significantly higher in respondents who reached or exceeded the SAS-SV cut-off score compared to respondents who were not considered being smartphone addicted.

The aORs of reporting clinically relevant symptoms of depression or anxiety increased gradually as the amount of smartphone usage accumulated. Across all duration categories of smartphone use, the aORs of being screened positive for depression (3–4 h/day: aOR = 1.71 [95%CI: 1.12–2.63]; 5–6 h/day: aOR = 2.29 [95%CI: 1.46–3.58]; 7–8 h: aOR = 3.79; [95%CI: 2.06–6.96]; >8 h/day: aOR = 7.61 [95%CI: 3.58–16.16]) and anxiety (3–4 h/day: aOR = 1.70 [95%CI: 1.10–2.63]; 5–6 h/day: aOR = 2.39 [95%CI: 1.52–3.75]; 7–8 h: aOR = 2.71 [95%CI: 1.53–4.78]; >8 h/day: aOR = 5.11 [95%CI: 2.69–9.71]) differed significantly from the reference category. At a level of 5–6 h/day, the aOR of reporting clinically relevant symptoms of insomnia (aOR = 1.81; [95%CI:1.03–3.15]) was significantly increased compared to low-users. At this level, there was also a significant increase in the odds of reporting higher levels of distress (aOR: 1.98 [95%CI: 1.25–3.12]), lower well-being (aOR = 2.25 [95%CI: 1.43–3.54]), and loneliness (aOR: 2.11 [95%CI: 1.31–3.39]) in comparison to low-users. For the category 7–8 h/day, the aORs of almost all mental health measures differed significantly from the reference category. At this level, the odds of being screened positive for disordered eating differed significantly from the reference category (0–2 h/day) (aOR = 2.12 [95%CI: 1.19–3.78]). Alcohol abuse is the only mental health indicator that was not related to the amount of daily smartphone use. Accordingly, for most mental health measures, smartphone use of at least five hours per day was significantly associated with poorer mental health than usage of two hours per day or less.

## 4. Discussion

More than one-third of adolescents and young adults reported PSU in Austria (33.3% of males, 39.0% of females). Since the estimated prevalence of smartphone addiction was about 18% for Europe [2], the rate of our respondents categorized as smartphone addicted by the SAS-SV seems rather high. PSU was associated with more frequent mental health symptoms, such as depressive or anxiety symptoms, symptoms of eating disorder, or alcohol abuse. However, in this study, PSU was not associated with loneliness, insomnia, distress, well-being, or physical activity. Furthermore, screen time was closely linked to PSU; however, screen time per se appeared to have a stronger association with mental health than PSU itself. In this context, we must point to the fact that about half of the respondents reported using their smartphones more than five hours per day, and about a fifth reported a minimum amount of seven hours per day.

The results of this study are in line with a large-scale Korean study based on over 50.000 adolescents. Woo and colleagues showed that smartphone screen time was problematic from 2 to 4 h of daily use depending on the purpose a smartphone was used for and above 4 h per day independent of the purpose [41]. This study also confirmed the findings of previous studies from Austria using only single-item scales to assess screen time [20,21,22].

Interestingly, not PSU but intensive screen time (≥5 h/d) was associated with distress, low well-being, or loneliness. From a minimum of 7 h/day, the aORs of almost all mental health problems (except for symptoms of alcohol abuse) were increased compared to low-users reporting a maximum of 2 h/day. We also found a dose–response manner in which the likelihood of reporting relevant symptoms of depression and anxiety gradually increased as the amount of smartphone use accumulated. Throughout the current survey, the purpose of smartphone use (e.g., educational, vocational, or leisure time) was not addressed, nor whether smartphone use added to further sources of screen time throughout the day. However, the results indicated an increase in the odds of depressive and anxiety symptoms from a minimum of three hours per day and an increase in the odds of insomnia symptoms, distress, low well-being, and loneliness from a minimum of 5 h per day. The daily amount of screen time was more strongly associated with these variables than PSU itself. However, this could also be due to the dichotomous categorization of the PSU (below SAS-SV cut-off vs. SAS-SV cut-off and above) and should not be overinterpreted.

We further point to the finding that more than a third (37.7%) of our respondents described themselves as being strongly lonely and that the amount of smartphone use was associated with an increased aOR of reporting being strongly lonely. Social isolation is contrasted with loneliness as an objective entity, with loneliness being described as the emotional perception of being isolated [42,43]. Considering the smartphone as a device which enables users to get into contact with others, our finding seems surprising. However, studies indicate that being virtually connected does not automatically prevent loneliness. Researchers concluded that virtual contacts do not satisfy social needs like direct interactions would [44]. Problematic internet use [9] or the amount of social media use [45] are associated with an increase in reported loneliness or perceived social isolation. Regarding smartphone use, a study revealed that lonelier respondents reported different smartphone usage patterns than individuals who described themselves as not lonely. Besides other variables, loneliness was associated with a higher amount of smartphone usage, with the purpose of use (i.e., social media vs. communication apps) and with a less frequent use of the smartphone [46]. With regard to PSU, a study on Chinese students concluded that loneliness predicted smartphone addiction [47]. On the other hand, research results also suggested that the regulated use of smartphones may also go along with a decrease in loneliness [48].

The association of PSU with depression and anxiety has been well documented before [3,7,8]. There are fewer studies in the literature about the relation between PSU and alcohol abuse or symptoms of eating disorders, especially in adolescents. A study including Chinese students for instance found that smartphone addiction was associated with symptoms of anxiety, depression, disturbed sleep as well as disordered eating and dysfunctional eating habits [49]. A study conducted by Domoff and colleagues [50] concluded that smartphone addiction correlated with difficulties regarding emotion regulation and different dysfunctional eating habits (e.g., restricted food intake and uncontrolled eating), food addiction, as well as their subjects’ body fat rate. In their sample, dysfunctional emotion regulation mediated between PSU and disordered eating [50]. However, with regard to PSU, a complex interplay between different variables like screen-based sedentary behavior, the purpose of smartphone use, and content-related variables (e.g., use of applications and platforms) must be considered. With respect to the main purpose of smartphone usage, especially the use of social media, platforms that focus on appearance have been associated with body dissatisfaction [51]. Nevertheless, there might be differing mechanisms responsible for the relation between the use of sites or the consumption of content and eating habits. A recently published international survey on adolescents found that the amount of daily smartphone screen time and the use of special social media sites are associated with different behaviors intended to change one’s weight (i.e., weight loss, weight gain, or no change) [52].

Some study results indicate an association between PSU and alcohol consumption [53,54,55]. A study conducted on university students found a significant association between problematic smartphone use (SAS-SV) and alcohol consumption [55]. A study on Swiss men also found associations of excessive smartphone use with alcohol intake. They, however, did not examine adolescents and did not use standardized instruments to assess symptoms of alcohol abuse. According to their results, problematic smartphone usage (SAS-SV) was associated with risky alcohol consumption (defined as the consumption of a minimum of six drinks of alcohol at least once per month and per one occasion) [54]. According to a Korean study, alcohol intake is associated with an increased likelihood of smartphone addiction in adolescents [53]. However, throughout their study, alcohol consumption was assessed with one single question, asking if they drank alcohol (yes/no).

With regard to symptoms of eating disorders within our sample, two important aspects must be mentioned at this point. Firstly, the screening tool SCOFF was primarily intended to assess symptoms associated with anorexia nervosa and bulimia nervosa [31]. Regarding symptoms of eating disorders, we did not assess other additional variables like body mass index (BMI) or (dys-)functional eating habits. Secondly, since we did not assess the purpose of smartphone usage, potential differences in the rates of disordered eating with respect to the main purpose of use (e.g., use of social media, listening to music, gaming, etc.) remain unclear.

Recently, recommendations on the duration of recreational screen time (including but not restricted to smartphone use) for children and adolescents were published [56]. Based on available research results, the authors recommend a maximum of 1–2 h of screen time per day for younger adolescents (12–16 years) and about 2 h per day for older adolescents (16–18 years) during their leisure time, regardless of the type of screen used (e.g., smartphone, tablet, television, etc.). Problematic aspects of high amounts of smartphone use are also related to sedentariness [14,15] and decreased physical activity and physical fitness [16,17,18,19]. Our results also indicate an association between physical inactivity and symptoms of depression, symptoms of anxiety, distress, and low well-being. 

Due to the complex interplay between extensive or problematic smartphone use, physical inactivity and mental health, the reduction in smartphone screen time or the regulated use of smartphones, as well as increased levels of physical activity, could serve as initial points of a self-reinforcing cycle [8,57,58]. Therefore, promoting recreational physical activity as well as providing information about favorable or functional smartphone use patterns are two important aspects within the public health discourse.

The further examination of causal relationships between mental health indicators and dysfunctional technology usage patterns is still a promising subject of research. For instance, longitudinal studies could examine causal links between (dys-)regulated smartphone usage and mental health indicators. Experimental designs for regulated smartphone usage patterns could further improve our understanding of the mechanisms linking dysfunctional technology use and mental health indicators.

### Limitations

Some aspects limit the generalizability of the present results and must be discussed. Firstly, the survey was conducted as a cross-sectional study; therefore, we duly underline that the nature of our study design does not allow for causal interpretations. Secondly, female respondents were overrepresented, as less than one-fifth of the participants identified as male or diverse. Thirdly, mental health outcomes were assessed via self-report screening tools, so bias regarding socially acceptable responses and bias due to possible impairments in the ability to introspect cannot be ruled out. Fourthly, we cannot rule out the possibility that individuals affected by mental health problems are more likely to participate in online surveys about mental health, so the presented results might have been affected by self-selection bias. Consequently, this convenience sample is not representative for gender, region, or educational levels. Fifthly, our results might have been affected by response bias. We cannot rule out that answers were confounded by factors like social desirability (i.e., regarding physical activity levels or the amount of smartphone usage) or inattention while answering the mental health questionnaires. Sixthly, two aspects also touch on the main behavioral aspects of interest: smartphone use and physical activity. Physical activity was assessed by using a single question asking about active days per week. We did not assess the types of physical activity our participants engage in. More specifically, we cannot draw any conclusions about the type or intensity of physical activity and its association with mental health. Another limitation regards the purpose of smartphone use. Our survey did not cover how respondents typically use their smartphones (e.g., gaming, social media, listening to music, watching videos, or educational purposes). Therefore, we cannot conclude if different patterns of smartphone use are associated with different mental health outcomes.

## 5. Conclusions

In sum, PSU is prevalent and associated with more frequent mental health symptoms, such as depressive or anxiety symptoms, symptoms of eating disorder, and alcohol abuse. However, intensive screen time seems to have a greater impact than PSU itself, and PSU is not associated with loneliness, distress, well-being, or physical activity. High amounts of smartphone usage (≥5 h/day) are associated with symptoms of depression, anxiety, and insomnia, as well as lower well-being and increased distress and loneliness. Reduced smartphone screen time and increased levels of physical activity seem to be two important starting points to improve mental health. Regarding smartphone usage, conclusions about dysfunctional smartphone use patterns and their potential risks with respect to mental and physical health, as well as suggestions for time limitations, should be drawn from research results to provide the general population with information about responsible smartphone use.

## Figures and Tables

**Table 1:** Sample characteristics of the total sample (N = 913) as well as their association with problematic smartphone use (SAS-SV; N = 881). Adjusted odds ratios and their ±95% confidence intervals are displayed.

Demographic Variables	Categories	Frequencies	PSU (N = 881)
		N = 913	aOR (±95%CI)
Age: Med [IQR]		17 [15; 18]	0.92 (0.83–1.03)
Gender: n (%)	Female	752 (82.4%)	Reference
	Male	129 (14.1%)	0.92 (0.60–1.41)
	Diverse	32 (3.5%)	-
Migration Background: n (%)	No	750 (82.1%)	Reference
	Yes	163 (17.9%)	1.01 (0.69–1.49)
Education: n (%)	Higher vocational education	390 (42.7%)	Reference
	Academic secondary school	333 (36.5%)	1.09 (0.78–1.51)
	University, university of applied sciences	81 (8.9%)	1.69 (0.92–3.10)
	(Pre-/intermediate) vocational education, apprenticeship	65 (7.1%)	0.55 (0.30–1.02)
	Other	44 (4.8%)	1.30 (0.65–2.62)
Smartphone Use: n (%)	≤2 h/day	125 (13.7%)	Reference
	3–4 h/day	340 (37.2%)	**3.59 (1.96–6.60)**
	5–6 h/day	267 (29.2%)	**6.96 (3.76–12.89)**
	7–8 h/day	95 (10.4%)	**13.38 (6.55–27.36)**
	>8 h/day	86 (9.4%)	**9.85 (4.74–20.46)**
Physical Activity: n (%)	Yes	792 (86.7%)	Reference
	No	121 (13.3%)	1.04 (0.67–1.60)
Loneliness: n (%)	Not lonely	142 (15.6%)	Reference
	Moderately lonely	427 (46.8%)	1.13 (0.73–1.75)
	Strongly lonely	344 (37.7%)	1.26 (0.80–1.99)

Note: Med = median, IQR = interquartile range; Migration Background (1st or 2nd generation); Physical Activity: yes = minimum of 60 min/day on at least 1 day per week up to a maximum of 7 days per week, no = 0 active days per week; PSU = problematic smartphone usage, being categorized as smartphone addicted by the SAS-SV (yes or no). On the right-hand side, aORs for the association of demographic variables with PSU. The data of diverse respondents were excluded from the analysis, since the SAS-SV is only provided with cut-off scores for male and female respondents; therefore, N = 881. Significant results are displayed in bold.

**Table 2:** Adjusted odds ratios and their ±95% confidence intervals for physical inactivity compared to physical activity, SAS-SV outcomes (below cut-off vs. cut-off and above), and different categories of smartphone use compared to ≤2 h/day.

	Physical Activity (N = 913)	PSU (N = 881)	Daily Amount of Smartphone Use (N = 913)			
	Inactivity vs. Activity	Below Cut-Off vs. Cut-Off and Above	3–4 h/day vs. ≤2 h/day	5–6 h/day vs. ≤2 h/day	7–8 h/day vs. ≤2 h/day	≥8 h/day vs. ≤2 h/day
	aOR (±95%CI)	aOR (±95%CI)	aOR (±95%CI)	aOR (±95%CI)	aOR (±95%CI)	aOR (±95%CI)
PHQ-9	**2.48** **(1.48–4.16)**	**1.46** **(1.06–2.01)**	**1.71** **(1.12–2.63)**	**2.29** **(1.46–3.58)**	**3.79** **(2.06–6.96)**	**7.61** **(3.58–16.16)**
GAD-7	**1.74** **(1.13–2.68)**	**1.86** **(1.37–2.51)**	**1.70** **(1.10–2.63)**	**2.39** **(1.52–3.75)**	**2.71** **(1.53–4.78)**	**5.11** **(2.69–9.71)**
ISI	1.29 (0.84–2.00)	1.04 (0.74–1.45)	1.27 (0.73–2.21)	**1.81** **(1.03–3.15)**	**2.76** **(1.45–5.27)**	**4.99** **(2.57–9.71)**
SCOFF	1.40 (0.91–2.17)	**1.55** **(1.13–2.11)**	1.29 (0.84–1.98)	1.51 (0.97–2.36)	**2.12** **(1.19–3.78)**	**2.73** **(1.43–5.20)**
CAGE	1.02 (0.63–1.64)	**1.71** **(1.19–2.45)**	1.22 (0.72–2.07)	1.07 (0.62–1.87)	1.20 (0.60–2.37)	1.59 (0.79–3.22)
PSS-4	**2.02** **(1.32–3.10)**	1.08 (0.80–1.46)	1.44 (0.93–2.24)	**1.98** **(1.25–3.12)**	**2.22** **(1.26–3.92)**	**4.68** **(2.48–8.85)**
WHO-5	**3.25** **(2.03–5.21)**	1.10 (0.81–1.49)	1.25 (0.80–1.94)	**2.25** **(1.43–3.54)**	**2.99** **(1.67–5.36)**	**4.54** **(2.36–8.74)**
Loneliness Scale	1.34 (0.88–2.02)	1.16 (0.86–1.57)	1.02 (0.64–1.64)	**2.11** **(1.31–3.39)**	**2.43** **(1.36–4.34)**	**2.26** **(1.23–4.15)**

Note: aOR = odds ratio; 95%CI = 95% confidence interval; depressive symptoms (PHQ-9); anxiety symptoms (GAD-7); insomnia symptoms (ISI); symptoms of disordered eating (SCOFF); symptoms of alcohol abuse (CAGE); higher levels of distress (PSS-4); low well-being (WHO-5); loneliness (Loneliness Scale: not/moderately lonely vs. strongly lonely). On the left-hand side, aORs for physical inactivity (reference group: physical activity, 1–7 active days per week, a minimum of 60 min/day). On the right-hand side, aORs across the categories of smartphone usage (h/day) compared to ≤2 h/day. In the middle, aORs for problematic smartphone use (SAS-SV; below cut-off vs. cut-off and above). The data of diverse respondents were excluded from the analysis, since the SAS-SV is only provided with cut-off scores for male and female respondents. Significant results are displayed in bold.

## Data Availability

The datasets generated during the current study are available from the corresponding author upon reasonable request.
